# The Influence of Recreational Substance Use in TMS Research

**DOI:** 10.3390/brainsci10100751

**Published:** 2020-10-18

**Authors:** Claudia V. Turco, Sarah O. Arsalan, Aimee J. Nelson

**Affiliations:** Department of Kinesiology, McMaster University, Hamilton, ON L8S 4L8, Canada; turcocv@mcmaster.ca (C.V.T.); arsalans@mcmaster.ca (S.O.A.)

**Keywords:** transcranial magnetic stimulation, caffeine, alcohol, nicotine, cannabis

## Abstract

(1) Background: Transcranial magnetic stimulation (TMS) approaches are widely used to study cortical and corticospinal function. However, responses to TMS are subject to significant intra-and inter-individual variability. Acute and chronic exposure to recreational substances alters the excitability of the sensorimotor system and may contribute to the variability in TMS outcome measures. The increasing prevalence of recreational substance use poses a significant challenge for executing TMS studies, but there is a lack of clarity regarding the influence of these substances on sensorimotor function. (2) Methods: The literature investigating the influence of alcohol, nicotine, caffeine and cannabis on TMS outcome measures of corticospinal, intracortical and interhemispheric excitability was reviewed. (3) Results: Both acute and chronic use of recreational substances modulates TMS measures of excitability. Despite the abundance of research in this field, we identify knowledge gaps that should be addressed in future studies to better understand the influence of these substances on TMS outcomes. (4) Conclusions: This review highlights the need for TMS studies to take into consideration the history of participant substance use and to control for acute substance use prior to testing.

## 1. Introduction

**Figure 1:**
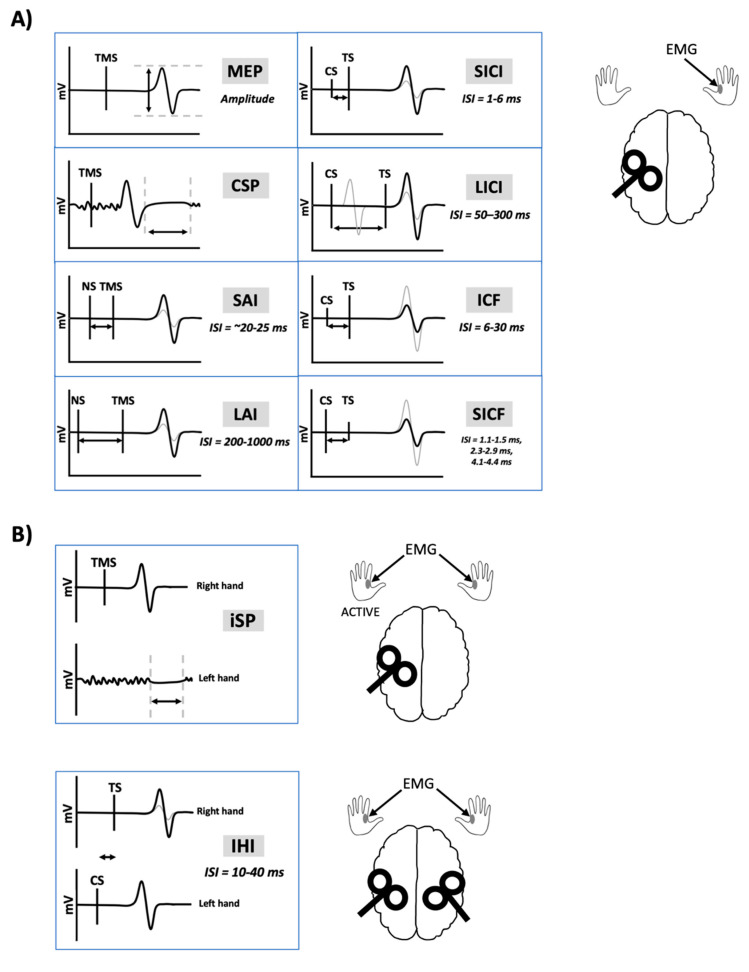
Transcranial magnetic stimulation-electromyography (TMS-EMG) outcome measures. Black lines indicate traces following a single pulse of TMS. Grey lines indicate traces following nerve stimulation (NS) and TMS paired together or following a conditioning stimulus (CS) and test stimulus (TS) pair. (**A**) Unilateral measures. TMS delivered to the left motor cortex results in a motor-evoked potential (MEP) recorded from a muscle in the right-hand using EMG. Delivering TMS during isometric contraction of the right-hand muscle leads to an interruption of voluntary contraction known as the cortical silent period (CSP). Short-latency afferent inhibition (SAI) and long-latency afferent inhibition (LAI) occurs when electrical peripheral NS is delivered prior to the TMS pulse, at interstimulus intervals (ISIs) of 20–25 ms or 200–1000 ms, respectively. Short-interval intracortical inhibition (SICI) is measured when a subthreshold CS is delivered 1–6 ms prior to a suprathreshold TS. The resulting MEP is inhibited, compared to the MEP obtained following the TS alone. Long-interval intracortical inhibition (LICI) is measured when a suprathreshold CS is delivered 50–300 ms prior to a suprathreshold TS, leading to inhibition of the MEP. Intracortical facilitation (ICF) is measured when a subthreshold CS is delivered 6–30 ms prior to a suprathreshold TS, leading to facilitation of the MEP. Short-interval intracortical facilitation (SICF) is measured when a suprathreshold CS is delivered 1.1–1.5 ms, 2.3–2.9 ms, or 4.1–4.4 ms prior to a subthreshold TS, leading to facilitation of the MEP. (**B**) Transcallosal measures. Delivering TMS to the left motor cortex during isometric contraction of the left-hand muscle leads to an interruption of voluntary contraction known as the ipsilateral silent period (iSP). Interhemispheric inhibition (IHI) is measured when a suprathreshold CS is delivered to the right motor cortex prior to a suprathreshold TS delivered to the left motor cortex, leading to inhibition of the MEP. Short-latency IHI (SIHI) occurs at ISIs of ~10 ms and long-latency IHI (LIHI) occurs at ISIs of ~40 ms.

Transcranial magnetic stimulation (TMS) has been extensively used to non-invasively probe the motor system in healthy and clinical populations to study the neural mechanisms of human movement and evaluate neuroplasticity. TMS paradigms are used to acquire measurements of corticospinal, intracortical and transcallosal excitability (Figure 1). Importantly, exogenous substances are capable of influencing the physiological state at the time of testing and may change cortical and corticospinal excitability as measured by TMS-evoked responses. Exogenous substances include recreational drugs such as nicotine, alcohol, cannabis, caffeine and other controlled substances. It is common for TMS studies to enroll university students as participants out of convenience. However, consumption of recreational substances such as alcohol [1], nicotine [2], and cannabis [3] peaks in young adults between the ages of 18–29.

Safety guidelines for TMS delivery suggest screening participants for consumption of alcohol and drugs that lower seizure thresholds [4]. However, there are no official guidelines regarding the screening of other common, recreational substances including cannabis, nicotine and caffeine. At present, it is unclear whether or how short-and long-term use of these substances are capable of inducing changes in neuronal excitability that is reflected in TMS measures or their variability. In this review, we consider research investigating acute and chronic use of recreational substances on TMS outcome measures for the purpose of identifying knowledge gaps that, if filled, may lead to new guidelines to improve the reliability and safety of TMS research. In this review, our use of the term “chronic use” refers to those with current or previous addiction or substance abuse. The review focuses on recreational substance use of alcohol, cannabis, caffeine and nicotine, and their impact on measures of corticospinal and cortical excitability.

## 2. Methods

A literature search was conducted using PubMed and EMBASE electronic databases from 1980 through August 2020. Separate searches were conducted for each substance of alcohol, cannabis, nicotine, and caffeine. The following search terms were used:

1. (transcranial magnetic stimulation) AND ((alcohol) or (alcoholism) or (ethanol));
2. (transcranial magnetic stimulation) AND ((nicotine) or (tobacco));
3. (transcranial magnetic stimulation) AND (caffeine);
4. (transcranial magnetic stimulation) AND ((cannabis) or (THC) or (marijuana)).

### Inclusion/Exclusion Criteria

Selected studies were required to meet the following inclusion criteria:

1. Studies assessed one or more of the following TMS measures: motor-evoked potential (MEP), resting motor threshold (RMT), active motor threshold (AMT), short-interval intracortical inhibition (SICI), intracortical facilitation (ICF), short-interval intracortical facilitation (SICF), long-interval intracortical inhibition (LICI), interhemispheric inhibition (IHI), short-latency afferent inhibition (SAI), long-latency afferent inhibition (LAI), cortical silent period (CSP), ipsilateral silent period (iSP), or TMS-evoked electroencephalography (EEG) potentials.
2. Primary research articles (i.e., original research) only.
3. Article was written in English.

**Figure 2:**
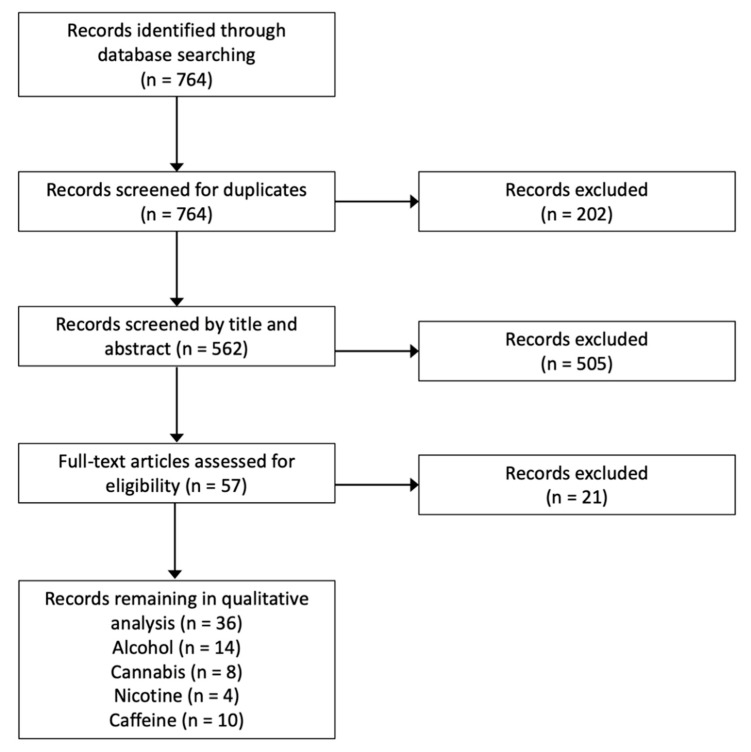
Preferred Reporting Items for Systematic Reviews and Meta-Analyses (PRISMA) diagram.

Studies assessing the influence of neuroplasticity-inducing protocols (e.g., repetitive transcranial magnetic stimulation, rTMS; paired associative stimulation, PAS; transcranial direct current stimulation, tDCS) on cravings or symptoms relating to chronic substance use were excluded as this was outside the scope of this review. For reviews on this topic, refer to Gorelick et al. [5], Hanlon et al. [6], Hauer et al. [7], or Mostafavi et al. [8]. Further, studies assessing the influence of substance use on the magnitude of corticospinal change following neuroplasticity-inducing protocols were excluded. Refer to Figure 2 for the Preferred Reporting Items for Systematic Reviews and Meta-Analyses (PRISMA) flow diagram. Details of all studies considered in this review are found in Table S1.

## 3. Alcohol

### 3.1. Acute Effects of Alcohol

Table 1 shows the effects of alcohol on TMS measures. There is a general consensus that acute alcohol intake facilitates gamma aminobutyric acid (GABA) neurotransmission and reduces glutamatergic neurotransmission [9,10]. Acute ethanol exposure increases GABA-mediated chloride-ion currents [11,12,13], increases cortical GABA-mediated inhibition [14], and facilitates the effects of GABA_A_ receptor (GABA_A_R) agonists [15]. In contrast, ethanol inhibits N-methyl-D-aspartate (NMDA) receptor activity [16,17] and reduces glutamate release [18,19,20].

Acute ethanol intake does not induce changes in RMT [21,22] or AMT [21], suggesting that ethanol does not modulate the excitability of the lowest threshold neurons in the primary motor cortex (M1). Furthermore, several studies demonstrate no effect of ethanol intake on MEPs [21,22,25]. However, it is not known if ethanol impacts the variability in MEPs.

The TMS findings from Ziemann et al. [21] reflect the consensus that acute alcohol intake facilitates GABAergic neurotransmission and reduces glutamatergic neurotransmission. A single dose of ethanol administered to healthy individuals increased SICI [21], suggesting an upregulation of GABA_A_R activity [33], and reduced ICF [21], suggesting a downregulation of NMDA receptor activity [33]. Further, ethanol increased the CSP [21,23], which may reflect an increase in GABA_B_ receptor (GABA_B_R) [33] and/or GABA_A_R activity [34]. Acute ethanol intake reduced the TMS-evoked N100 potential following stimulation of the dorsolateral prefrontal cortex (DLPFC) [25] and M1 [26]. The N100 potential is modulated by both baclofen (a GABA_B_R agonist) and benzodiazepines (positive allosteric modulators of the GABA_A_R) [35]. Several studies suggest that upregulation of GABA_B_R activity may serve as a homeostatic mechanism to regulate the sensitivity of GABA_A_Rs to ethanol [36,37,38]. Therefore, modulation of the N100 potential likely reflects the complex mechanisms through which ethanol exerts its pre-and postsynaptic effects via GABA_B_Rs and GABA_A_Rs, respectively. To our knowledge, no study has assessed the influence of alcohol intake on the amplitude of the N45 potential. However, given that the N45 potential is reflective of GABA_A_R activity similar to SICI [35], this may suggest that alcohol intake would reduce the N45 potential. Finally, SICF is reduced following ethanol consumption [22]. This suggests that ethanol exerts its effects at the level of I-wave generating interneurons, which are thought to be regulated by GABAergic mechanisms [39].

Alcohol reduced short-latency interhemispheric inhibition (SIHI) in healthy females, but not males [24]. This aligns with evidence that alcohol has a larger physiological impact in females. Females are more susceptible to alcohol-related cognitive impairments than males [40,41,42,43], likely because females reach higher peak blood alcohol levels than males even when adjusting for difference in weight [44]. Circulating levels of sex steroid hormones also interact with ethanol. Specifically, testosterone injection inhibits ethanol-induced impairments in spatial memory [45] and estradiol increases the sensitivity of dopaminergic neurons in the ventral tegmental area (VTA) to ethanol [46]. Therefore, future research investigating the effect of alcohol on TMS should consider the influence of biological sex.

#### Summary

In summary, findings from the TMS literature appear to indicate that acute intake of alcohol increases SICI and CSP, while reducing ICF, SICF, SIHI, and the TMS-evoked N100 potential (Table 1). However, the acute administration of alcohol does not modulate MEP amplitude, and so does not appear to influence the corticospinal excitability.

### 3.2. Chronic Effects of Alcohol

Chronic alcohol exposure has the opposite effect of acute exposure characterized by a reduction in GABAergic neurotransmission and an increase in glutamatergic neurotransmission [9,10,47]. Chronic alcohol exposure reduces the sensitivity of GABA_A_Rs [48,49,50], the density of cortical GABA_A_Rs carrying α1-and α5-subunits [51,52] and blunts the effect of GABA_A_R agonists [37,53]. Furthermore, chronic alcohol exposure increases NMDA receptor-mediated excitatory post-synaptic potentials and glutamate release [19]. The dichotomy between the acute and chronic effects of alcohol may be due to physiological changes in the dopaminergic reward pathway that occur during the transition to chronic alcohol consumption [54]. Acutely, ethanol stimulates dopamine release in a dose-dependent fashion within the nucleus accumbens (NAc) [55,56,57,58,59]. Chronically, ethanol lowers circulating dopamine levels by increasing the rate of dopamine re-uptake in the NAc and the sensitivity of the D2 autoreceptor, which acts to inhibit dopamine release [60].

Alcohol-dependents exhibit reduced RMT, AMT, and MEPs compared to healthy controls [30,32]. In contrast, other studies report no chronic-related effects on RMT, AMT or MEPs [23,28,31]. Importantly, Naim-Feil et al. [30] recruited participants on the basis that they had successfully completed a detoxification program within the past two years. This indicates that while thresholds are not altered in chronic alcoholics, the physiological changes resulting from detoxification induced changes in thresholds. However, it is unknown if and for how long these physiological changes persist beyond two years.

In young adults who exhibited alcohol-dependence in adolescence, TMS-evoked N45 potentials were larger compared to controls [31]. This suggests an upregulation of GABA_A_R activity [35], which does not follow the expected chronic effects of alcohol [9,10,47]. Alcohol-dependence during a stage of development may have led to different physiological changes compared to the effect of alcohol-dependence in adulthood.

Several studies have reported no difference in SICI between alcohol-dependents and controls, suggesting that chronic alcohol intake does not modulate GABA_A_R activity within M1 [23,28,30]. The underlying effect of alcoholism on different subunits of the GABA_A_Rs may explain why alcohol dependence leads to an increase in the N45 potential but no change in SICI, which are both modulated by GABA_A_R activity. The N45 potential is increased by agonists to the α1-subunit [35], while SICI likely reflects GABA_A_Rs with α2/3-subunits [33,61,62]. This may suggest that alcohol-dependence has a physiological effect only on specific subunits of the GABA_A_R (i.e., the α1 subunit). LICI is reduced within the DLPFC of alcohol-dependents compared to healthy controls, which suggests hyperexcitability of the prefrontal cortex as a result of reduced GABA_B_R activity [30].

The chronic effects of alcohol on SAI and LAI have yet to be investigated. Unlike SICI, SAI is likely modulated by GABA_A_Rs containing the α1-subunit [62]. Similar to SAI, LAI is also modulated by the GABA_A_R agonist lorazepam [63]. If chronic alcohol intake specifically alters functioning of GABA_A_Rs with the α1-subunit, it can be hypothesized that it would lead to an alteration in SAI and LAI.

Chronic ethanol exposure does not have an effect on CSP [23,28,30]. However, these studies may have been underpowered to expose an effect due to the relatively small sample sizes used [23,28,30]. Individuals at high-risk for alcohol dependence exhibit shorter CSP and iSP [27], but no difference in RMT or the % maximum stimulator output (%MSO) to evoke a 1 mV amplitude MEP in comparison to low-risk individuals [29]. This suggests that those predisposed to alcohol dependence may have inherited an imbalance of excitation/inhibition. Future studies should investigate if these predisposed changes in physiology also lead to differential effects of acute alcohol intake on cortical function. Evidence for a potential effect comes from in vitro studies, where rodent strains bred for high ethanol sensitivity exhibit greater effects of ethanol on GABA-receptor chloride conductance compared to low-alcohol sensitive rodents [9,64,65].

One important consideration is the dependency on alcohol at the time of testing. Compared to both controls and chronic alcoholics, individuals with alcohol-withdrawal syndrome exhibit increased ICF [28], which may reflect hyperexcitability as a result of chronic exposure. However, there is no difference in SICI between controls and alcoholics when individuals are not tested during withdrawal [23,30]. Overall, the effects of chronic alcohol consumption appear to be dependent upon an individual’s physiological state at the time of testing.

#### Summary

Based on the findings reviewed herein, chronic alcohol consumption appears to modulate intracortical circuitry underlying SICI, ICF, SICF and CSP (Table 1). Overall, the effects of chronic alcohol consumption appear to be dependent upon an individual’s physiological state at the time of testing—whether individuals are alcohol-dependents with versus without withdrawal symptoms in adulthood, were previous dependents in adolescence, recently completed detoxification, or have a familial history of alcohol dependence. This widespread effect clearly demonstrates the considerable impact that long-term consumption of alcohol has on neurophysiology.

## 4. Cannabis

According to the World Health Organization, ~2.5% of the global population consumes cannabis annually. Accompanying its legalization, the prevalence of cannabis use has risen by 15% in Canada [66] and 25% in the United States [67]. Long-term cannabis use is a risk factor for the development of schizophrenia [68] and neurocognitive impairments [69], and leads to structural changes such as increased cortical thickness [70,71,72] and cerebellar volume [73,74], and reduced hippocampal [75,76,77] and prefrontal cortex volume [77]. As such, it is essential to further our understanding of the impact that cannabis has on TMS measures.

In the brain, cannabis exerts its effect on cannabinoid type 1 receptors (CB1Rs), which presynaptically modulate GABA, glutamate, dopamine and acetylcholine levels. Animal studies show that CB1R agonists enhance dopaminergic neurotransmission in the basal ganglia [78,79] and mesolimbic pathway [80], leading to a reduction in prefrontal cortical activity [80]. CB1R agonists inhibit the release of GABA from pyramidal neurons in the prefrontal cortex [81], hippocampus and the VTA [80,82,83,84], and reduce glutamatergic neurotransmission in the prefrontal cortex [85], hippocampus [86,87], and NAc [88]. Finally, cannabinoids reduce acetylcholine release in the prefrontal cortex and hippocampus [89,90].

In humans, acute administration of delta-9-tetrahydrocannabinol (∆-9-THC) increases striatal dopamine release [91,92,93]. Cannabis addiction follows the neurobiological model of addiction proposed by Koob and Volkow [94], marked by dysregulation of neural circuitry within the mesocorticolimbic dopamine system, amygdala and prefrontal cortex [95]. Chronic cannabis exposure leads to a reduction in dopamine synthesis capacity in the striatum [96,97]. GABA_B_R agonists and GABA reuptake inhibitors enhance the symptoms from acute ∆-9-THC intake [98,99], and long-term cannabis use reduces GABAergic function in the anterior cingulate cortex (ACC) [100,101]. Cannabis users also exhibit reduced glutamate levels in the basal ganglia [102], prefrontal cortex [103] and ACC [100,101]. Overall, evidence from animal models and humans clearly demonstrates diverse neurobiological effects of cannabis across brain regions.

The effects of cannabis on TMS measures can be seen in Table 2. In a case study with one participant comorbid for Tourette’s syndrome and attention deficit hyperactive disorder (ADHD), ∆-9-THC increased CSP length and SICI but did not affect RMT or the stimulation intensity to evoke a MEP of 1 mV [104]. This suggests that ∆-9-THC modulates intracortical inhibitory circuits within M1. However, this was observed in a single participant and may not generalize to larger sample sizes. In addition, the patient studied was consuming additional medications, making it harder to interpret the results [104]. The acute effects of cannabis on TMS measures should be tested in future studies.

Fitzgerald et al. [105] reported that both heavy and light cannabis users show reduced SICI compared to controls, which has since been replicated [109]. Similarly, Martin-Rodriguez et al. [106] recently showed that SICI is reduced in daily cannabis users and participants with cannabis abuse disorder compared to healthy controls. This is in line with other research suggesting that cannabis inhibits GABAergic neurotransmission [80,81,82,100,101]. In contrast, Flavel et al. [108] reported no difference in SICI between cannabis users and non-users. Discrepancies between these findings may relate to methodological differences. Specifically, Flavel et al. [108] tested a sample of individuals who were comorbid for chronic use of alcohol in addition to cannabis, whereas the other studies controlled for comorbid alcohol abuse [105,106,109]. Furthermore, the sample size used in Flavel et al. [108] was likely underpowered to detect a group difference in SICI. Neither study reported a difference in RMT, MEPs, CSP, or ICF between users and non-users [105,106,108,109]. Furthermore, there were no reported group differences in AMT [105,106] or LICI [105,109]. These findings suggest that, within M1, long-term cannabis use modulates GABA_A_R, but not GABA_B_R or NMDA receptor function.

It is notable that all TMS studies showing an effect of cannabis abuse on SICI also reported that the cannabis group had significantly fewer years of education compared to controls [105,107,109]. The detrimental effects of cannabis on education outcomes may be influenced by other factors such as childhood adversity, family structure, and socioeconomic status [113,114]. It is currently unknown if intracortical inhibition is related to years of education, intelligence, or other sociocultural factors like socioeconomic status. Further longitudinal research is required to determine if education is a significant modifier of SICI.

Other studies investigating the chronic effects of cannabis use have been conducted in participants diagnosed with schizophrenia, as one third of schizophrenia patients report using cannabis daily [115]. These individuals experience more severe psychotic symptoms in response to ∆-9-THC [116], whereas the antipsychotic properties of cannabidiol (CBD) improves symptoms of schizophrenia [117,118]. Schizophrenia patients comorbid for cannabis abuse demonstrate increased ICF and reduced SICI compared to schizophrenia patients that do not use cannabis [107]. In contrast, Goodman et al. [109] reported that schizophrenia patients dependent on cannabis show increased SICI compared to non-users. This may be attributable to the inclusion of a sample of schizophrenia patients with a longer disease duration and a stricter criterion for defining cannabis abuse.

Sativex, an oromucosal spray containing ∆-9-THC and CBD, is commonly administered to multiple sclerosis (MS) patients for pain relief. MS patients treated with Sativex show no change in AMT, RMT or MEPs [110,111], or sensorimotor excitability as shown by no change in SAI or LAI [110]. Russo et al. [110] showed that Sativex reduces SICI and increases ICF, while Leocani et al. [111] showed no change in SICI or ICF after Sativex treatment.

In a recent study, Calabrò et al. [112] assessed the influence of cannabis on motor function following robot-aided gait training. Specifically, two groups of MS patients underwent 6 weeks of gait training along with administration of a THC:CBD oromucosal spray added onto ongoing oral antispastic therapy or oral antispastic therapy only. Those treated with THC:CBD demonstrated greater increases in MEP amplitude and greater decreases in SICI and ICF within the APB muscle following gait training. However, when obtained from the tibialis anterior (TA) muscle, a similar magnitude of MEP increase, SICI and ICF decrease were observed in both groups following gait training. These changes in cortical and corticospinal excitability were also accompanied by changes in function. Specifically, those treated with THC:CBD showed greater improvements in muscle stiffness, functional independence, ambulation, quality of life, and global disability following gait training compared to the group not treated with THC:CBD. Therefore, these results show that cannabis administration can potentiate rehabilitative outcomes following motor training.

### Summary

Overall, chronic cannabis use appears to have no effect on corticospinal excitability but does impact SICI. Specifically, chronic exposure to cannabis consistently modulates SICI in healthy individuals and those diagnosed with schizophrenia. However, the effects of cannabis on other TMS measures including SAI, LAI, SICF, IHI and iSP have yet to be investigated. In addition, there is a gap in the knowledge regarding the acute effects of cannabis, within both long-term users and non-users.

## 5. Nicotine

Nicotine induces an inward depolarizing current by acting on nicotinic acetylcholine receptors (nAChRs) on one of two binding sites: *α*4*β*2 and *α*7-subunits [119]. Low doses of nicotine act on both binding sites, while higher doses act predominantly on *α*7-containing nAChRs due to the rapid desensitization of the *α*4*β*2-containing nAChRs [120,121]. Acutely, nicotine-induced activation of nAChRs leads to depolarization of glutamatergic neurons expressing the *α*7-nAChRs and GABAergic neurons expressing non-*α*7-nAChRs [122]. However, repeated nicotine exposure leads to the desensitization of non-*α*7-nAChRs on GABAergic neurons with no significant desensitizing effect on *α*7-nAChRs on glutamatergic neurons [120,122]. This translates to net excitation of mesolimbic dopaminergic neurons [120,122]. However, the net result of nicotine on excitation/inhibition balance varies across brain regions [123], leading to inhibition or disinhibition of pyramidal neurons [124]. Chronic exposure also leads to reduced cortical perfusion [125], reduced microstructural integrity of cerebral white matter [126], and increased dendritic arborization within M1 [127].

TMS can be used to gauge the effects of nicotine on motor function, although the reported results are mixed (Table 3). In healthy non-smokers, acute nicotine intake had either not changed SICI and SAI [128] or increased SICI and SAI [129]. SAI is reduced by muscarinic antagonists [130] and increased by acetylcholinesterase inhibitors [131,132], demonstrating its involvement in the cholinergic system. Therefore, increased SAI following nicotine intake likely reflects the upregulation of nAChR activity, whereas increased SICI may reflect the nAChR-modulation of GABAergic neurotransmission [62]. Orth et al. [128] may not have shown a significant effect of nicotine on SICI or SAI because of the low dose of 2 mg nicotine that was administered, whereas Grundey et al. [129] administered a higher dose of 16 mg. Furthermore, nicotine was administered in the form of gum [128] compared to a nicotine patch [129]. Approximately half of the nicotine administered in gum form is absorbed, reducing its effectiveness as an intervention compared to a nicotine patch [133,134,135]. Regardless, these studies showed no effect of nicotine on thresholds, MEP, CSP, SICF or ICF in non-smokers [128,129].

In smokers, acute nicotine intake also had no effect on MEPs [138,139]. Alternatively, acute nicotine intake increased ICF in smokers and did not modulate SAI or SICI [129]. These findings may be explained by the evidence mentioned above, where chronic exposure to nicotine may lead to the desensitization of non-*α*7 nAChRs that modulated GABAergic neurotransmission, while glutamatergic neurotransmission remains elevated [120].

Relative to non-smokers, smokers exhibited no difference in thresholds [129,136,137], iSP [137], SICF [129], SICI [129,136], LAI or LICI [136], but did show increased SAI [129] and reduced ICF [129,136]. Studies have reported that MEPs in smokers are either elevated [129,137], reduced [136], or not different to controls [136,140]. Lavendar et al. [140] likely did not see an effect of chronic nicotine exposure on MEPs as they only tested the %MSO required to evoke a 1 mV MEP while Grundey et al. [129] showed that, across a range of stimulation intensities, smokers only exhibit elevated MEPs at high intensities of 150% RMT only. Similarly, Lang et al. [136] showed that resting MEPs obtained at 110%, 120% or 140% RMT were not different between groups, while only MEPs obtained during active contraction were reduced in smokers compared to non-smokers. Additionally, CSP is either increased [136] or not different from non-smokers [137,140]. Discrepancies across studies may be a result of the different contraction levels maintained during CSP acquisition, which varied from 30–50% maximum voluntary contraction (MVC) [136], 50% MVC [137] or 10% maximum voluntary force (MVF) [140].

### Summary

Acute nicotine intake appears to modulate SAI and SICI in non-smokers with no effects on corticospinal excitability. In smokers, chronic nicotine exposure modulates corticospinal excitability, CSP, SAI and ICF compared to non-smokers. However, SICI, LICI, SICF, iSP and LAI appear to be similar between smokers and non-smokers. As such, screening for both recent and chronic nicotine intake is important to consider in future TMS research.

## 6. Caffeine

Health Canada recommends 400 mg/day as an upper limit for caffeine intake in adults, as moderate caffeine intake of 400 mg/day is not associated with toxicity or adverse health effects [141]. Nevertheless, caffeine is considered to be a psychostimulant, and is known to affect brain function even when consumed at levels below the recommended upper limits. At low doses (20–200 mg), caffeine enhances attention, reaction time, and motor coordination [142,143,144,145]. At high doses (250–500 mg), caffeine increases unpleasant feelings of tension, irritability, and anxiousness, and reduces the amplitude of alpha and beta waves recorded with EEG [146].

Caffeine exerts its psychomotor effects by inhibiting adenosine receptors, specifically the A1 and A2A receptor subtypes [147,148,149]. A1 receptors are expressed in the hypothalamus, hippocampus, basal ganglia, and cortex; A2A receptors are localized in the striatum, NAc and olfactory bulb [150]. Caffeine also induces a presynaptic increase in glutamate release and reduces miniature excitatory post-synaptic currents via the blockade of α-amino-3-hydroxy-5-methyl-4-isoxazoleproprionic acid (AMPA) ionotropic glutamate receptors [151]. However, it is unlikely that regular caffeine intake in humans would have a significant effect on glutamate ionotropic receptors, as this effect was found under extreme conditions of caffeine toxicity [151]. Research suggests dopamine’s involvement in eliciting the psychostimulant effects that follow caffeine intake. The motor and discriminative stimulus effects of caffeine are diminished when dopamine is depleted or when dopamine receptors are blocked [152,153]. Moreover, the presence of caffeine increases dopaminergic transmission, which is linked to an increase in arousal and motivation, coupled with enhanced serotonin release and increased post-synaptic serotonergic input [150].

The effect of caffeine on TMS measures are shown in Table 4. First, acute caffeine intake has no effect on threshold [154,155,156,157,158]. Multiple studies have reported no change in resting MEPs following caffeine intake, at intensities ranging from 100–150% RMT [154,155,156,157,158,159,160,161]. Bowtell et al. [162] also found no change in resting MEPs following caffeine intake, while MEPs obtained during maximal contraction were potentiated by caffeine. However, Specterman et al. [163] found that 68 mg of caffeine increased resting MEPs from 30–90 min after intake. This effect was potentiated when participants were administered Lucozade, an energy drink containing 68 g of caffeine and 46 g of glucose. Notably, these finding were obtained from a small sample of four and six participants, respectively. Alternatively, caffeine has been demonstrated to potentiate post-exercise facilitation of MEPs on multiple occasions [158,159,161].

Acute caffeine intake has no reported effect on SAI and LAI [158], or SICI [154,157,158]. One study reported a reduction in SICI following caffeine intake, although a similar finding was also observed in the placebo condition [155]. This suggests that the change in SICI was not due exclusively to the effects of caffeine.

Most studies have reported no effect of caffeine on ICF [154,155,157]. However, Concerto et al. [158] found a decrease in ICF after administration of a sugar-free energy drink, which contained caffeine as the main active ingredient. The authors attributed this effect to taurine, which is present within energy drinks in significant concentrations. Taurine is a free amino acid that modulates GABA_A_R activity [164,165] and glutamatergic neurotransmission [165,166,167]. In a follow-up study, Infortuna et al. [168] found that taurine alone was not capable of modulating ICF. The reduction in ICF reported by Concerto et al. [158] may be attributed to time of testing, as TMS measures were acquired 45 min following ingestion of the energy drink. Studies reporting no effect of caffeine on ICF performed testing at least 1 h following caffeine administration [154,155,157].

CSP is either reduced [155,156,157] or not affected by caffeine [154,158,162]. Studies reporting a decrease following caffeine intake obtained CSP at low TMS intensities of 110% RMT [156,157]. Alternatively, studies reporting no change in CSP used higher intensities of 120–175% AMT [154,162] or 150% RMT [158]. Indeed, Cerqueira et al. [156] showed that only CSP obtained at 110% RMT and not 150% RMT was reduced following caffeine intake. Mesquita et al. [155] revealed a reduction in CSP following caffeine intake at a higher intensity of 130% RMT, although this was acquired in the soleus muscle. CSP length increases with increasing TMS intensity [169]. It is possible that the neuromodulatory effects of caffeine are not capable of inducing a change in CSP at higher TMS intensities.

### Summary

Based on these findings from the literature, caffeine does not appear to have a significant modulatory effect on motor thresholds, MEPs, SAI, LAI, SICI or LICI. However, studies acquiring CSP or ICF should consider restricting caffeine prior to testing. At present, it is unknown if caffeine leads to a change in interhemispheric measures of IHI or iSP, and it is currently unknown whether cortical excitability is different between habitual versus non-habitual coffee consumers.

## 7. Current Gaps in Knowledge

There are several notable gaps in the research reviewed. Notably, as seen in Table 1 through Table 4, the acute and chronic effects of recreational substances on several measures are unknown. For example, the acute and chronic effects of alcohol on measures such as LICI, LAI, SAI, IHI and iSP are unknown. Furthermore, the effects of chronic cannabis use on SAI, LAI, SICF, IHI and iSP have yet to be tested. Therefore, until further investigation can be undertaken, studies employing all of these TMS measures should screen participants for recent recreational substance use.

The time-course of substance effects on TMS measures has not been thoroughly investigated. Future studies examining the effect of acute substance intake on TMS measures should include multiple post-intake measurements to determine how long the substance exerts an observable effect of motor physiology. Furthermore, it would also be useful to have information about blood plasma levels of substances over time, and how this related to TMS measures. For drugs consumed orally, the variability in gastric emptying due to food constituents in the stomach may contribute to variability in the time course of effects.

In extension, the time course of variation in TMS measures within chronic substance users, users undergoing withdrawal, and those in recovery is unknown. Studies examining the effect of chronic exposure on TMS measures should include samples at different points of withdrawal or recovery to determine if and when motor function normalizes. For example, the studies reviewed in Table 4 instructed participants to refrain from caffeine intake for some time before TMS testing in order to better identify the effects of the administered caffeine dose. A side effect of prolonged abstinence from caffeine is the emergence of withdrawal symptoms [170]. It is unknown whether corticospinal or cortical excitability is altered during periods of caffeine withdrawal in habitual coffee consumers. Concrete testing is needed to fully elucidate whether the physiological state of withdrawal or recovery has a differential impact on TMS measures compared to periods of chronic substance use.

There has been limited investigation into the interaction between biological sex and recreational substance use on TMS measures. However, biological sex appears to be a determinant of the brain’s response to cannabis and nicotine. Female cannabis users have lower glutamate and glutamine levels in the dorsal striatum [171] and impaired frontal dopaminergic neurotransmission [172] compared to controls. Male cannabis users do not show this effect [171,172]. It is unknown whether differences in neurotransmitter profiles between male and female cannabis users are reflected within TMS outcome measures. Furthermore, females are less sensitive to nicotine [173] and are less responsive to nicotine replacement therapies [174]. This suggests that nicotine induces differential changes in physiological state between biological sexes. Future TMS studies should determine whether there is an interaction between acute or chronic nicotine or cannabis exposure and biological sex on outcome measures.

Fitzgerald et al. [105] divided cannabis consumers into groups of light and heavy consumers, and showed that both groups experienced reduced SICI. This indicates that infrequent consumption can still lead to persisting changes in cortical excitability. However, the cross-sectional design of this study prevented the quantification of the average dose consumed by participants. Importantly, no studies have assessed the dose-dependent effects of substance use on TMS measures, except for Cerquiera et al. [156] who found that both 200 mg and 400 mg of caffeine reduced CSP. Therefore, future studies should examine the dose-response relationship between recreational substances and TMS metrics, which would more suitably tested with an intervention study.

We can attribute 60–70% of the variance in nicotine dependence to genetic factors [175]. There are a number of molecular variants that have been linked to nicotine dependence [176]. For example, individuals expressing variations in CHRNA4, CHRNA5, and CHRNB4, which code for nAChRs subunits, exhibit greater nicotine dependence [177,178,179], greater number of cigarettes smoked per day [180,181], and increased vulnerability to smoking [182]. Studies should investigate if individuals expressing these variants, or variants related to dependence on other recreational substances, show altered cortical excitability relative to individuals expressing the wildtype alleles.

Specifically related to cannabis research, an important consideration for future studies is the effect that different strains of cannabis have on neurophysiological function. The two main ingredients of cannabis are ∆-9-THC, the psychoactive component, and CBD, the non-psychoactive component. These components may have differential effects on brain function [183,184]. Specifically, ∆-9-THC intake reduced striatal activation while CBD increased striatal activity during a word retrieval task [183]. ∆-9-THC and CBD had similar opposing effects on task-related amygdala, hippocampal, temporal and occipital activation [183]. Future studies should account for the differential effects of CBD and ∆-9-THC in TMS research.

Substance abuse is often comorbid with other psychiatric conditions, as exemplified by the comorbidity of schizophrenia and cannabis use. Further, those with alcoholism may present comorbidities for depression [185], bipolar disorder [186], or Wernicke–Korsakoff syndrome [187]. Due to the prevalence of comorbidities in those with substance use disorders, it is important that future TMS studies assessing the chronic effects of recreational substances take care to screen participants for existing comorbidities. Furthermore, studies should also continue to assess the interactions between substance use and comorbid psychiatric disorders, and how this changes neurophysiological function.

It is unknown whether the reliability of TMS measures is impacted by recreational substances. While intake of recreational substances did not significantly change TMS outcome measures in some cases, we cannot conclude that they had no effect on the variability of these measures. Finally, eight of the 21 intervention studies reviewed did not include a placebo control (see Table S1). As such, the results of these studies should be interpreted with caution. Future studies should continue to employ placebo-controlled and blinded study designs to increase outcome validity.

## 8. Conclusions

According to the studies reviewed herein, both acute and chronic use of recreational substances appear to modulate the excitability of the motor system, reflected by a change in TMS outcome measures. Overall, we note that most studies suffered from a small sample size and did not report power or sample size calculations. Heterogeneity stemming from participant demographics and parameters used to acquire TMS outcome measures also limits our interpretation of these studies. However, the results do suggest that there is a need for future studies to take into consideration the history of substance use and to control for acute substance use at the time of testing.

Although there is not enough information to provide definitive screening guidelines, our assessment of the literature suggests that this information can still be implemented in TMS screening tools. However, preliminary screening guidelines may include excluding participants with a history of chronic recreational substance use and acute use within the 24 h prior to testing, even though the influence of abstinence duration following intake still needs to be determined. Finally, the information provided in this review allows for a retrospective assessment of datasets demonstrating a causal effect of substance use on TMS measures.

## Figures and Tables

**Table 1:** Effects of alcohol on TMS measures.

Study	AMT	RMT	MEP	CSP	iSP	SAI	LAI	SICI	ICF	SICF	LICI	SIHI	LIHI	N100	N45	Notes
Ziemann et al. [21]	○	○	○	▲	–	–	–	▲	▼	–	–	–	–	–	–	Acute intake
Ziemann et al. [22]	–	○	○	–	–	–	–	–	–	▼	–	–	–	–	–	Acute intake
Conte et al. [23]	–	○	○	▲	–	–	–	–	–	–	–	–	–	–	–	Acute intake in controls
	–	○	–	○	–	–	–	○	○	–	–	–	–	–	–	Alcoholics vs. controls
Hoppenbrouwers et al. [24]	–	–	▼	–	–	–	–	–	–	–	–	▼*	–	–	–	Acute intake; * significant in females only
Loheswaran et al. [25]	–	–	○	–	–	–	–	–	–	–	–	–	–	▼*	–	Acute intake in alcoholics; * acquired in DLPFC
Kähkönen et al. [26]	–	–	–	–	–	–	–	–	–	–	–	–	–	▼*	–	Acute intake; * acquired in M1
Muralidharan et al. [27]	–	○	–	▼	▼	–	–	–	–	–	–	–	–	–	–	High-vs. low-risk for alcohol dependence
Nardone et al. [28]	○	○	–	○	–	–	–	○	▲	–	–	–	–	–	–	AWS vs. alcoholics and controls
Muralidharan et al. [29]	–	○	○	–	–	–	–	–	–	–	–	–	–	–	–	High-vs. low-risk for alcohol dependence
Naim-Feil et al. [30]	▼	▼	▼	○	–	–	–	○	○	–	▼*	–	–	–	–	Alcoholics vs. controls; * LICI acquired in DLPFC
Kaarre et al. [31]	–	○	–	–	–	–	–	–	–	–	–	–	–	–	▲	Heavy alcohol use in adolescence vs. controls
Quoilin et al. [32]	–	–	▼	–	–	–	–	–	–	–	–	–	–	–	–	Alcoholics vs. controls

▲ increase; ▼decrease; ○ indicates no change; − indicates did not assess; * refers to stipulations outlined in the right-hand column; AMT: active motor threshold; AWS: Alcohol Withdrawal Syndrome; CSP: cortical silent period; DLPFC: dorsolateral prefrontal cortex; ICF: intracortical facilitation; iSP: ipsilateral silent period; LAI: long-latency afferent inhibition; LICI: long-interval intracortical inhibition; LIHI: long-latency interhemispheric inhibition; M1: primary motor cortex; MEP: motor-evoked potential; N100: TMS-evoked electroencephalography potential at 100 ms latency; N45: TMS-evoked electroencephalography potential at 45 ms latency; RMT: resting motor threshold; SAI: short-latency afferent inhibition; SICF: short-interval intracortical facilitation; SICI: short-interval intracortical inhibition; SIHI: short-latency interhemispheric inhibition.

**Table 2:** Effects of cannabis on TMS measures.

Study.	AMT	RMT	MEP	CSP	iSP	SAI	LAI	SICI	ICF	SICF	LICI	SIHI	LIHI	Notes
Hasan et al. [104]	–	○	○	▲	–	–	–	▲	–	–	–	–	–	Acute intake
Fitzgerald et al. [105]	○	○	○	○	–	–	–	▼	○	–	○	–	–	Heavy and light cannabis users vs. non-users
Martin-Rodriguez et al. [106]	○	○	○	–	–	–	–	▼	–	–	–	–	–	CUD and daily cannabis users vs. non-users
Wobrock et al. [107]	–	○	–	–	–	–	–	▼	▲	–	–	–	–	Schizophrenia cannabis users vs. non-users
Flavel et al. [108]	–	○	○	○	–	–	–	–	–	○	○	–	–	Cannabis users vs. nonusers
Goodman et al. [109]	–	○	–	○	–	–	–	▲	○	–	○	–	–	Schizophrenia cannabis users vs. non-users
	–	○	–	○	–	–	–	▼	○	–	○	–	–	Control cannabis users vs. nonusers
Russo et al. [110]	○	○	○	○	–	○	○	▲	▼	–	–	–	–	MS patients on 1 month of Sativex
Leocani et al. [111]	–	○	○	–	–	–	–	○	○	–	–	–	–	MS patients on 1 month of Sativex
Calabrò et al. [112]	–	–	▲	–	–	–	–	▼	▼	–	–	–	–	MS patients on 6 weeks of Sativex + gait training

▲ increase; ▼decrease; ○ indicates no change; – indicates did not assess; CUD: cannabis use disorder; MS: multiple sclerosis.

**Table 3:** Effects of nicotine on TMS measures.

Study	AMT	RMT	MEP	CSP	iSP	SAI	LAI	SICI	ICF	SICF	LICI	SIHI	LIHI	Notes
Grundey et al. [129]	○	○	○	–	–	▲	–	▲	○	○	–	–	–	Non-smokers after acute intake
	○	○	○	–	–	○	–	○	▲	○	–	–	–	Smokers after acute intake
	○	○	○	–	–	○	–	○	▼	○	–	–	–	Smokers vs. non-smokers
Orth et al. [128]	○	○	–	○	–	○	–	○	○	–	–	–	–	Non-smokers after acute intake
	○	○	–	○	–	○	–	○	○	–	–	–	–	Tourette’s after acute intake
Lang et al. [136]	○	○	▼	▲	–	▲	○	○	▼	–	○	–	–	Smokers vs. non-smokers
Khedr et al. [137]	○	○	▲	○	○	–	–	–	–	–	–	–	–	Smokers vs. non-smokers

▲ increase; ▼decrease; ○ indicates no change; – indicates did not assess.

**Table 4:** Effects of caffeine on TMS measures.

Study	AMT	RMT	MEP	CSP	iSP	SAI	LAI	SICI	ICF	SICF	LICI	SIHI	LIHI	Notes
Kalmar et al. [159]	–	–	○	–	–	–	–	–	–	–	–	–	–	Acute intake
Orth et al. [154]	○	○	○	○	–	–	–	○	○	–	–	–	–	Acute intake
Specterman et al. [163]	–	–	▲	–	–	–	–	–	–	–	–	–	–	Acute intake
Cerqueira et al. [156]	–	○	○	▼	–	–	–	–	–	–	–	–	–	Acute intake
de Carvahlo et al. [157]	–	○	○	▼	–	–	–	○	○	–	○	–	–	Acute intake
Concerto et al. [158]	–	○	○	○	–	○	○	○	▼	–	–	–	–	Acute intake
Hanajima et al. [160]	–	–	○ *	–	–	–	–	–	–	–	–	–	–	Acute intake; * between-subject comparison
Kalmar et al. [161]	–	–	○ *	–	–	–	–	–	–	–	–	–	–	Acute intake; * trending increase after caffeine intake, but not significant
Bowtell et al. [162]	–	–	▲ *	○	–	–	–	–	–	–	–	–	–	Acute intake; * caffeine only potentiated MEPs obtained during maximal contraction
Mesquita et al. [155]	○	–	○	▼	–	–	–	○ *	○	–	–	–	–	Acute intake; * SICI reduced in caffeine and placebo condition

▲ increase; ▼decrease; ○ indicates no change; – indicates did not assess; * refers to stipulations outlined in the right-hand column.

## Supplementary Materials

The following are available online at https://www.mdpi.com/2076-3425/10/10/751/s1: Table S1: TMS studies investigating the effects of recreational substance use.
