# Supplementary material for: The Influence of Recreational Substance Use in TMS Research
Source: Brain Sci. 2020 Oct 18;10(10):751. doi: 10.3390/brainsci10100751 (PMC7603156; doi:10.3390/brainsci10100751)
Supplement: Supplementary file 1 [file brainsci-10-00751-s001.pdf]

Table S1. TMS studies investigating the effects of recreational substance use.

Substance	Study	Sample	Study Design	Target Muscle	Dose Administered/Use History	Detection and/or Levels in the Body
Alcohol	Ziemann et al. [17]	6 healthy (4M, 26.8 ± 3.6y)	I	ADM	0.7L wine (12% vol. ethanol)	Mean BAC: 0.73 ± 0.1ml/L
	Ziemann et al. [18]	6 healthy (26.3 ± 2.6y)	I	ADM	0.7L wine (12% vol. ethanol)	N/A
	Conte et al. [19]	13 alcoholics (11M, 49 ± 6y), 10 controls (7M, 50 ± 4y)	I	FDI	Alcoholics: 18y of consumption with 13 drinks/day<br>Controls: administered 24g (males) or 12g (females) of ethanol	Alcohol dependents: negative BAC in breath test<br>Controls: 30 ± 6.05mg/dL (males) and 32 ± 6.0mg/dL (females)
	Muralidharan et al. [23]	15 high-risk & 15 low-risk for alcohol dependence (all M, 20.5 ± 4.2y)	CS	OP	High risk: alcohol-naïve subjects with family history of alcohol dependence<br>Low risk: alcohol-naïve subjects without family history of alcohol dependence	N/A
	Hoppenbrouwer et al. [20]	22 healthy (10M, 22.5 ± 0.9y)	I (PC)	APB	300mL solutions of alcohol/orange juice mix until BAC reached 0.5%	BAC of 0.5%
	Nardone et al. [24]	13 AWS (8M, 48.4y), 12 alcoholics (8M, 47.6y), 15 controls (8M, 46.8y)	CS	FDI	AWS: diagnosis in accordance to DMS-IV<br>Alcoholics: 13.4 drinks/day for 17y	N/A
	Muralidharan et al. [25]	16 high-risk & 12 low-risk for alcohol dependence (12-25y)	CS	FDI	High risk: alcohol-naïve subjects with family history of alcohol dependence<br>Low risk: alcohol-naïve subjects without family history of alcohol dependence	N/A
	Naim-Feil et al. [26]	12 alcoholics in post-detox phase (8M, 40.1	CS	FDI	Alcoholics: 15 drinks/day for 16y, tested 145 days post	N/A

		± 13.4y), 14 controls (7M, 31.1 ± 5.3y)			successful completion of a detox program	
	Kaarre et al. [27]	27 heavy long-term alcohol use in adolescence (11M, 23-29y), 25 controls (12M, 23-29y)	CS	APB	10y heavy alcohol use from adolescence to young adulthood. No withdrawal symptoms at time of testing.	N/A
	Loheswaran et al. [21]	15 healthy alcohol drinkers (10M, 33.4 ± 7.5y)	I (PC, SB)	APB	At least 1 heavy drinking episode (2h period of 5 drinks in males or 4 drinks in females) within the last month. Administered alcoholic beverages until BAC > 17.4mM	Mean BAC: 23.6 ± 4.1mM
	Quoilin et al. [28]	20 alcoholics (9M; 51.1 ± 6.9y), 20 controls (9M; age-matched)	CS	FDI	Alcoholics: 14.6y of alcohol dependence, mean 19.9 units (1 unit = 10g ethanol) of alcohol per day, abstinent for 17-20 days during assessment	N/A
	Kähkönen et al. [22]	10 healthy (all M; 25 ± 3.7y)	I	ADM	0.8g/kg ethanol	N/A
Cannabis	Fitzgerald et al. [99]	25 heavy users (20M; 28.6 ± 9.5y), 17 light users (11M; 25.1 ± 6.9y), 19 non-users (13M; 28.9 ± 9.1y)	CS	APB	Heavy users: used cannabis >7x/week<br>Light users: used cannabis 1-4x/week	All heavy and 6/17 light users had a positive urine test for cannabis. 16/20 heavy users had a quantifiable THC plasma level (2ng/ml at least)
	Wobrock et al. [101]	12 SCZ with comorbid cannabis abuse (10M; 24.4y), 17 SCZ non-users (11M; 33.6y)	CS	FDI	Substance abuse/dependence assessed by the EuropASI and DSM-IV criteria, and defined as cannabis use for >20x in their lifetime, at least weekly consumption over a period of at least 12 weeks in the last 12 months	Negative urine test for alcohol, THC, hallucinogens, and opiates

Hasan et al. [98]	1 comorbid for TS & ADHD (M, 15y)	I	FDI	Initial dose of 5mg Δ-9-THC, then increased to 15mg/day for 9 weeks	Blood levels: Δ-9-THC was <1.0ng/mL, 11-hydroxy-δ-9-THC was <1.0ng/mL, 11-Nor-δ-9-THC-9-carbonate was 8.8ng/mL
Flavel et al. [102]	26 abstinent stimulant users (17M, 28 ± 7y), 9 cannabis users (6M; 23 ± 7y), 17 non-users (9M; 25 ± 7y)	CS	FDI	Stimulant users: use of stimulants on >5 occasions (ecstasy, methamphetamine, cocaine, pharmaceuticals) Cannabis users: use of cannabis on >5 occasions	Negative urine test for amphetamines, methamphetamines, ecstasy, cocaine, opioids or benzodiazepines. Participants with positive urine test for THC included if use was >12h before testing.
Goodman et al. [103]	12 SCZ with comorbid cannabis use (12M; 29.4 ± 8.4y), 11 SCZ non-users (7M; 38.5 ± 8.9y), 10 cannabis users (10M; 30.4 ± 7.4y), 13 non-users (10M; 35.5 ± 10.5y)	CS	APB	Cannabis dependence: daily use for at least 1y and smokes daily (minimum 5 cigarettes)	N/A
Martin-Rodriguez et al. [100]	14 CUD (all M; 23 ± 3y), 14 daily cannabis users (all M; 24 ± 3y), 15 controls (all M; 25 ± 3y)	CS	FDI	CUD: 5 ± 3y of cannabis use (>10x/week), daily cannabis use over previous 6 months, classified as CUD with CUDIT-R score of 13.9 ± 4 Daily users: 6 ± 3y of cannabis use (>10x/week), daily cannabis use over previous 6 months, classified as non-CUD with CUDIT-R score of 8.5 ± 4	Plasma Δ-9-THC: 5.3-6 ng/mL in daily users, 4.2-6.4 in CUD
Russo et al. [104]	30 MS patients (sex not reported, >18	I	APB	1 month of Sativex intake	N/A

		years, mean age not reported)				
	Leocani et al. [105]	43 MS patients (23M; 48 ± 8y)	I (PC, DB)	FDI	1 month of Sativex intake	N/A
	Calebro et al. [106]	Group A: 20 MS patients (10M; 52 ± 11y)<br>Group B: 20 MS patients (8M; 47 ± 8y)	CS (PC, SB)	TA/APB	Group A: 6 weeks treatment with RAGT and THC:CBD oromucosal spray in addition to normal oral antispastic therapy<br>Group B: 6 weeks of RAGT and oral antispastic therapy	N/A
Nicotine	Orth et al. [122]	9 TS (6M; 2 comorbid for ADHD, 1 comorbid for ADH & OCD, 31.3y), 10 controls (7M; 32.6y)	I	FDI	Nicotine gum (2mg)	Plasma nicotine: 4.4 ± 1.3ng/ml in controls and 4.2 ± 1.6ng/ml in TS
	Lang et al. [130]	Experiment 1: 12 smokers (10M, 25 ± 0.6y), 12 non-smokers (10M, 24 ± 0.7y)<br>Experiment 2: 19 smokers (13M, 24 ± 0.4y), 19 non-smokers (13M, 24 ± 0.4y)	CS	APB	Smokers: minimum 10 cigarettes/day for 4y (Fagerström score of 2.92 ± 0.4 in experiment 1, 3.19 ± 0.24 in experiment 2)	N/A
	Grundey et al. [123]	12 smokers (8M; 26 ± 4y), 12 non-smokers (6M; 25 ± 4y)	I	ADM	Smokers: minimum 10 cigarettes/day for 4y<br>Nicotine transdermal patch (16mg over 6h)	N/A
	Khedr et al. [131]	25 smokers (all M, 39.6 ± 15.1y), 25 non-smokers (all M, 42.2 ± 13.5y)	CS	FDI	Smokers: minimum 10 cigarettes/day for 3y	N/A

Caffeine	Kalmar et al. [153]	7 healthy (all M; 25 ± 5y)	I (PC, DB)	FDI	All participants self-reported caffeine intake <250 mg/week (low consumption)<br>Caffeine capsule (6mg/kg)	N/A
	Orth et al. [148]	11 healthy (7M; 32.9y, range 24–38)	I (PC, DB)	FDI	Decaf coffee with added 3mg/kg caffeine	Plasma caffeine: 0.95 ± 0.48µg/ml at 60min and 0.7 ± 0.36µg/ml at 120min
	Specterman et al. [157]	10 healthy (5M; 21-50y); 6 in Lucazode trials; 4 in control trials	I (PC)	Thenar	Lucozade trials: Lucozade, a sports drink containing 380ml water, 68g glucose and 46mg caffeine<br>Control trials: 380ml water, 380ml water with 68g glucose, or 380ml water with 46mg caffeine (over 3 sessions)	N/A
	Cerqueira et al. [150]	Experiment A: 18 healthy (9M; 28.4 ± 4.8y)<br>Experiment B: 6 healthy (4M; 28.8 ± 8.8y)	I (PC, DB)	ADM	Experiment A: 200mg caffeine capsule<br>Experiment B: 400mg caffeine capsule	Plasma caffeine: 3.85 ± 0.09µg/ml at 60min in experiment A, 10.41 ± 1.67µg/ml at 60min in experiment B
	de Carvalho et al. [151]	13 healthy (3M; 27.5 ± 3.3y)	I (PC, DB)	ADM	200mg of caffeine	N/A
	Concerto et al. [152]	14 healthy (6M; 31.2 ± 9y)	I (PC, DB)	APB	Sugar-free energy drink with 2mg/kg caffeine	N/A
	Mesquita et al. [149]	18 healthy (all M; 26.6 ± 3.1y)	I (PC, DB)	SOL	Caffeine capsule (6mg/kg)	8.51mg/L 60min after intake, 7.04mg/L after fatigue protocol
	Hanajima et al. [154]	12 healthy (6M; 44.8 ± 1.4y)	I (PC, DB)	FDI	200mg of caffeine	N/A
	Kalmar et al. [155]	8 healthy (all M; 22.5 ± 1.9y)	I (PC, DB)	VL	Caffeine capsule (6mg/kg)	N/A
	Bowtell et al. [156]	9 recreational athletes (all M; 26 ± 2.7y)	I (PC, DB)	VM	Caffeine capsule (6mg/kg)	N/A

ADHD: Attention-Deficit Hyperactive Disorder, ADM: abductor digiti minimi, APB: abductor pollicis brevis, AWS: alcohol-withdrawal syndrome, BAC: blood alcohol concentration, CBD: cannabidiol, CS: cross-sectional, CUD: cannabis use disorder, CUDIT-R: Cannabis Use Disorder Identification Test-Revised, DB: double-blind, ECR: extensor carpi radialis, FDI: first dorsal interosseous, I: intervention, MS: multiple sclerosis, N/A: not available, OP: opponens pollicis, PC: placebo-controlled, RAGT: robot-aided gait training, SB: single-blinded, SCZ: Schizophrenia, SOL: soleus, TAL tibialis anterior, THC: delta-9-tetrahydrocannabinol, TS: Tourette's Syndrome, VL: vastus lateralis, VM: vastus medialis.
